# Response of Root-Associated Bacterial Communities to Different Degrees of Soft Rot Damage in *Amorphophallus konjac* Under a *Robinia pseudoacacia* Plantation

**DOI:** 10.3389/fmicb.2021.652758

**Published:** 2021-07-08

**Authors:** Fei He

**Affiliations:** School of Modern Agriculture and Biotechnology, Ankang University, Ankang, China

**Keywords:** *Amorphophallus konjac*, bacterial community composition, Illumina high-throughput sequencing, root-associated bacteria, soft rot disease

## Abstract

Bacterial soft rot is a destructive disease that restricts the development of the konjac (*Amorphophallus konjac* K. Koch ex N.E.Br) industry. The objective of this study was to investigate how soft rot disease affects bacterial communities associated with the roots of konjac plants growing under a pure *Robinia pseudoacacia* plantation. Three sampling sites affected by different degrees of soft rot damage were selected based on the disease incidence [0%, non-diseased (ND); 4.2%, moderately diseased (MD); and 18.6%, highly diseased (HD)]. The variation in soil and root bacterial diversity and community composition among the sampling sites was determined by Illumina HiSeq sequencing of the V3–V4 hypervariable regions of the bacterial 16S rRNA gene. The results showed that the contents of soil organic matter and available nutrients (N, P, and K) increased with increasing damage degree, whereas higher damage degree resulted in lower soil pH and enzymatic activity (sucrase, urease, catalase, and polyphenol oxidase). The composition of root-associated bacterial communities differed among the three sampling sites. Proteobacteria was the most dominant bacterial phylum in all soil and root samples. *Pseudomonas*, *Bacillus*, *Rhizobium*, and *Streptomyces* were the most abundant in all samples from the ND sites, whereas *Pectobacterium carotovorum* and *Serratia* were predominant in the samples from the MD and HD sites. The abundance and alpha diversity of root-associated bacteria were significantly higher (*p* < 0.05) in the ND sites than in the diseased sites. The results suggested pronounced differences in the abundance, alpha diversity, and community composition of bacteria associated with the roots of konjac plants affected by different degrees of soft rot damage. Such differences in bacterial community structure were related to dynamic changes in soil variables, especially soil available potassium content, sucrase activity, and urease activity. Analysis of the dominant root-associated bacterial taxa offers an approach to predict the damage degree due to soft rot in konjac and provides evidence for the prevention of this soil-borne disease via microecological regulation.

## Introduction

Konjac (*Amorphophallus konjac* K. Koch ex N.E.Br) is a perennial, herbaceous species extensively planted in Southeast Asia and Africa (Vasques et al., 2008). The corm of konjac is rich in glucomannan, a neutral polysaccharide with excellent biocompatibility and biodegradability. Konjac glucomannan has been widely used in food, medicine, agriculture, and chemical engineering (Li et al., 2010). China is a major producer of konjac and has the world’s largest konjac production capacity (Wu et al., 2010; Qiu, 2013). Langao (Shaanxi Province) is a national key county of konjac industry in China (Song and Liu, 2018). The planting area of konjac in Langao reached 62.67 km^2^ in 2019, accounting for approximately one-sixth of the total planting area of konjac in Shaanxi Province (The State Council of the People’s Republic of China, 2019).

To make full use of limited land resources, konjac intercropping is applied in various plantations, such as *Robinia pseudoacacia*, *Juglans regia*, *Toxicodendron vernicifluum*, and *Hevea brasiliensis*. Among them, the yield of konjac under *R. pseudoacacia* plantations is the highest, with an average increase of 71.9% compared with the konjac yield in corn fields (He et al., 2015c). So far, the practice of planting konjac under *R. pseudoacacia* plantations has been widely adopted throughout China, including Zhen’an County in Shaaxi Province (He et al., 2014) and other regions in Yunnan Province (Zhang, 2017). Despite the yield increase in intercropping, development of the konjac industry in China is still negatively affected by soft rot, a devastating soil-borne plant disease caused by *Pectobacterium* spp. (Wu et al., 2012), *Dicyeya* spp. (Czajkowski et al., 2014; Wu et al., 2016), and *Enterobacter* spp. (Wu et al., 2011b).

Outbreaks of konjac soft rot disease have caused irreparable damage to the agroecosystem with serious economic losses and ecological destruction, including the added costs of labor management and disease control (Wu et al., 2011a). Therefore, many studies have been dedicated to identifying the pathogens of konjac soft rot and developing new control methods (Wu et al., 2011b, 2012, 2015; Chen, 2013). Biological control, for instance, using *Paenibacillus polymyxa* and *Pseudomonas fluorescens*, is considered to be a promising method for the control of konjac soft rot (Helmy et al., 2008; Chen, 2013). However, the effects of available biological agents or bioorganic fertilizers are unstable in practice due to the influence of abiotic and biotic factors. Particularly, the microecosystem formed by microbes in the roots and root-associated soil of konjac is a vital factor affecting the incidence of soft rot and regulating the efficacy of biological control (He and Cui, 2017).

The microbial diversity and community composition in the root zone of a crop is indicative of the present situation and development tendency of the soil ecosystem, which is crucial for crop health (He et al., 2015c). It has been found that agricultural practices, including crop variety selection (Guan et al., 2016), fertilizer application (Su et al., 2014), continuous cropping (Liu et al., 2019), interplanting, and rotation (Zhuang et al., 2009), can cause variation in soil microbial diversity, microbial abundance, and enzymatic activity. Moreover, a previous study using culture-dependent technique has shown that the community composition of root-associated bacteria and fungi differs between konjac growing fields with and without soft rot (He et al., 2018). However, little information is available on the response of soil and root bacterial communities to different degrees of soft rot damage in konjac. Additionally, the relationships between root-associated bacteria and soil variables in konjac growing sites affected by different degrees of soft rot damage remain poorly understood. Detangling these relationships is essential to identify the key factors influencing the bacterial community structure in the root zone of konjac.

In the present study, we hypothesized that different degrees of soft rot damage in konjac could affect the abundance, alpha diversity, and community composition of root-associated bacteria through altering soil variables. To verify the hypothesis, we compared soil physicochemical properties and enzymatic activity, as well as soil and root bacterial community characteristics, among three konjac growing sites affected by soft rot disease to different degrees. The results could provide supportive evidence for the prevention and control of konjac soft rot via microecological regulation.

## Materials and Methods

### Study Area

This study was conducted on konjac plants growing under a pure black locust (*R. pseudoacacia*) plantation located in Lishuya, a village in Langao County, Shaanxi Province, Northwest China. Langao (32°N and 108°E) is situated in the northern subtropical continental monsoon climate zone, with a mean altitude of 1,486 m and a high forest coverage of 78.6%. The mean annual temperature is 16°C, and the frost-free period lasts 242 days. The mean annual dryness index and precipitation are 0.37 and 1,033 mm, respectively. Precipitation is highly seasonal, with ∼80% of the annual precipitation occurring from May to October and a dry season of 6 months. The major soil type in the study area is brown clay (FAO-UNESCO, 1988).

### Experimental Design and Sample Collection

Three sampling sites were chosen in the study area based on the incidence and disease index of konjac soft rot (Supplementary Figure 1). One site was not infected with soft rot [non-diseased (ND)], while two sites were affected by soft rot to different degrees [moderately diseased (MD) and highly diseased (HD)]. All the sampling sites had been continuously planted with the same cultivar konjac (*A. konjac* K. Koch ex N.E.Br) for 2 years, and the current konjac plants were 7 months old. The canopy closure of *R. pseudoacacia* in each site was evaluated by an experienced forestry expert using a simple visual assessment technique (Jennings et al., 1999). Other information on the sampling sites was provided by the Bureau of Konjac Langao (Shaanxi, China).

In August 2017, three sampling plots (20 m × 20 m) were established at random in each of the ND, MD, and HD sites (NDS, MDS, and HDS). The MD plants were identified by maceration of corms, and the leaves were yellow on one side of the plants. The HD plants were identified by maceration and rotting of parenchymatous tissue in aboveground organs (stems and leaves) in addition to underground organs, which led to a total collapse of the aboveground parts of the plants (Wu et al., 2010). The incidence and disease index of konjac soft rot were calculated according to the method of Duan et al. (2018).

In each sampling plot, three 7-month-old konjac plants (70–90 cm tall) were selected at random for collecting soil and root samples simultaneously. After removal of leaf litter and stones on the soil surface (0- to 5-cm depth), root-associated soil samples (each ∼500 g) within 2 cm of the roots of selected konjac plants were collected by pulling the plant from the ground and shaking the roots gently (April and Keller, 1990). After that, fresh root samples of the same three konjac plants were obtained together with soil from the 5- to 30-cm depth. In total, three soil samples and three root samples per plot were collected separately. Subsamples were combined equally to give one composite sample per plot. The soil samples of the same three konjac plants were passed through a 2-mm mesh size sieve and then divided into two portions. One portion was stored at −80°C for DNA extraction, and the other portion was air-dried and stored at 25°C in resealable plastic bags until analysis for soil physicochemical properties and enzymatic activity. The root samples were washed five times with sterile water and then stored at −80°C prior to DNA extraction.

### Soil Physicochemical Analysis and Enzymatic Activity Assay

Soil pH was measured in 1:5 (w/v) soil–water suspensions (Bao, 2000) using a PHS-3C digital pH meter (Lida Instrument Factory, Shanghai, China). Soil organic matter (OM) was determined by oxidization with 0.4 M of potassium dichromate–concentrated sulfuric acid solution (Nelson and Sommers, 1982). The alkaline hydrolysis diffusion method was used to determine soil available nitrogen (AN) content (Song et al., 2018). Soil available phosphorus (AP) was extracted with 0.5 M of NaHCO_3_ for 20 min and quantified by molybdenum-antimony anti-spectrophotometry (Page, 1982). Soil available potassium (AK) was extracted with 1.0 M of ammonium acetate for 30 min and determined by flame photometry (Oyedele et al., 2008).

Soil sucrase activity was assayed using the 3,5-dinitrosalicylic acid colorimetric method and calculated on the basis of the amount of reducing sugar liberated (Guan et al., 1986). Soil urease activity was assayed using the indophenol blue colorimetric method, which involved an incubation step with 5% urea (substrate) in borate buffer solution (pH 10) for 24 h, subsequent extraction, and filtration with 2 M of KCl (Kandeler and Gerber, 1988). Soil catalase activity was assayed by titration with 0.1 M of potassium permanganate (Stepniewska et al., 2009). Polyphenol oxidase activity was analyzed using the catechol colorimetric method with 0.5% catechol as a substrate (Adamczyk et al., 2009).

### DNA Extraction and Illumina HiSeq Sequencing

Soil and root samples were homogenized in liquid nitrogen. Total genomic DNA was extracted from 500 mg of soil samples using the E.Z.N.A.^®^ soil DNA kit (Omega Bio-Tek, Doraville, GA, United States) following the manufacturer’s instructions. Total DNA extraction from 80 mg of root samples was performed using a plant DNA extraction kit (Tiangen Biotech, Beijing, China) according to the manufacturer’s protocol. A total of 18 samples, consisting of nine root samples and nine soil samples (three biological replicates with three technical replicates for each treatment), were subjected to total genomic DNA extraction. DNA concentration and purity were determined using a Smartspec^TM^ Plus spectrophotometer (Bio-Rad, Hercules, CA, United States), and the quality was monitored using 0.8% agarose gel electrophoresis.

The DNA extract was diluted to 1 ng⋅μl^–1^ and used as PCR template. The universal primers 319F (5′-ACTCCTACGGGAGGCAGCAG-3′) and 806R (5′-GGACTACHVGGGTWTCTAAT-3′) were used to amplify the V3–V4 hypervariable regions of the bacterial 16S rRNA gene (Zhou et al., 2011), which covered over 97% of the 16S gene sequences in the ribosomal database. Each primer included a linker sequence required for Illumina HiSeq 300-bp paired-end sequencing and a 12-bp heterogeneity spacer index sequence intended to reduce biases associated with low-diversity amplicon sequencing (Chopyk et al., 2017).

DNA samples were PCR-amplified in triplicate. The PCR mixture (25 μl) contained 13 μl of 2 × Taq Master Mix (CoWin Biotech, Beijing, China), 0.5 μl of each primer (10 μM; Invitrogen, Carlsbad, CA, United States), 1 μl of DNA template, and 10 μl of RNase-free water. The following thermal conditions were used: pre-denaturation at 95°C for 3 min; 37 cycles of degeneration at 95°C for 5 s, annealing at 58°C for 30 s, and extension at 72°C for 45 s; and a final extension step at 72°C for 10 min. Gel electrophoresis was performed to confirm successful amplification. Next, PCR products from the triplicate reactions were extracted from a 0.8% agarose gel and further purified using a universal DNA purification kit (Tiangen Biotech). The pellet of combined DNA was submitted to Beijing Biomics Biotechnology Institute (Beijing, China) for paired-end sequencing on the Illumina HiSeq 2500 Platform (Illumina Inc., San Diego, CA, United States).

### Sequence Data Processing and Analysis

Raw tags generated by Illumina sequencing were transformed into clean tags using FLASH v1.2.7 (Magoč and Salzberg, 2011), Trimmomatic v0.33 (Wang et al., 2012), and UCHIME v4.2 (Edgar et al., 2011). The clean sequences were clustered into operational taxonomic units (OTUs) based on a 97% similarity cutoff using Usearch v7.1 in the QIIME pipeline (v1.8.0; http://qiime.org/). Representative sequences for each bacterial OTU were chosen for alignment and taxonomic classification using the Ribosomal Database Project classifier (Release 11.5; http://rdp.cme.msu.edu/). Rarefaction curve and alpha diversity indices were analyzed based on the OTU table by random sampling to the minimum number of sequences in the samples. The alpha diversity of bacteria in each sample was evaluated in terms of species richness (Chao1 and ACE) and diversity (Shannon and Simpson indices), and the index values were calculated using QIIME v1.8.0 (Caporaso et al., 2010). All raw sequences were submitted to the Sequence Read Archive of the National Center for Biotechnology Information database under accession numbers SRR11490401–SRR11490418.

### Statistical Analysis

Significant differences between treatments were analyzed through a one-way analysis of variance (ANOVA) followed by least significant difference (LSD) tests (*p* < 0.05) using SPSS Statistics v22.0 (SPSS Inc., Chicago, IL, United States). Venn diagrams were created to visualize common and unique OTUs between samples (http://bioinformatics.psb.ugent.be/webtools/Venn/; Chen and Boutros, 2011). A heatmap of dominant bacterial genera (relative abundance > 1% of total sequences) was constructed using the package ‘‘pheatmap’’ in R v3.2.2^1^. Principal component analysis (PCA) was conducted based on the relative abundance of dominant and pathogenic bacterial genera from the three sampling sites using Canoco v5.0 (Centre for Biometry, Wageningen, Netherlands). Redundancy analysis (RDA) was used to estimate the multivariate correlations between bacterial community structure and soil variables associated with different degrees of konjac soft rot using Canoco v5.0.

## Results

### General Characteristics of Konjac Growing Sites

The three sampling sites had distinctly different incidences and disease index of konjac soft rot: 0 and 0 for ND, 4.2% and 1.4 for MD, 18.6% and 18.6 for HD. The three sites were located at similar altitudes (963–1,043 m) and slopes (29°–32°). All the sites were derived from the same original vegetation (*R. pseudoacacia*), with the canopy closure of 57–60%, average plant height of 81.8–85.5 cm, and planting density of 5.56–6.25/m^2^ (Table 1).

**TABLE 1:** Profile of three konjac growing sites in the study.

**Site**	**Altitude/m**	**Canopy**	**Slope**	**Average plant**	**Soil**	**Annual average**	**Planting**	**Incidence of**	**Disease**
		**closure/%**		**height/cm**	**texture**	**precipitation/mm**	**density/m^2^**	**soft rot/%**	**index**
ND	1,043	60	32°	85.5	Brown clay	1,033	6.25	0	0
MD	963	58	29°	82.7	Brown clay	1,033	5.56	4.2	1.4
HD	995	57	30°	81.8	Brown clay	1,033	6.25	18.6	18.6

*ND, MD, and HD represent the non-diseased, moderately diseased, and highly diseased konjac stands infected by soft rot disease, respectively.*

### Soil Physicochemical Properties and Enzymatic Activity

Soil pH decreased concomitantly with increasing degree of soft rot damage, and the lowest soil pH was observed in site HD (6.40). In contrast, soil OM, AN, AP, and AK contents all showed the opposite trend (Table 2) and reached the highest levels in site HD (28.38 g⋅kg^–1^, 127.36 mg⋅kg^–1^, 8.27 mg⋅kg^–1^, and 110.53 mg⋅kg^–1^, respectively). However, there were no significant differences in soil AN and AP contents between sites ND and MD (*p* > 0.05).

**TABLE 2:** Soil physicochemical properties in the three konjac growing sites affected by different levels of soft rot disease.

**Site**	**Soil pH**	**Soil organic matter**	**Alkaline-hydrolyzable nitrogen**	**Available phosphorus**	**Available potassium**
		**(OM; g⋅kg^–1^)**	**(AN; mg⋅kg^–1^)**	**(AP; mg⋅kg^–1^)**	**(AK; mg⋅kg^–1^)**
ND	6.63 ± 0.06a	21.66 ± 2.91c	106.35 ± 3.96b	5.14 ± 0.55b	70.83 ± 0.45c
MD	6.61 ± 0.10a	26.92 ± 3.53ab	116.05 ± 5.45b	6.22 ± 0.76b	91.67 ± 2.12b
HD	6.40 ± 0.06b	28.38 ± 2.30a	127.36 ± 3.91a	8.27 ± 0.81a	110.53 ± 0.83a

*ND, MD, and HD represent the non-diseased, moderately diseased, and highly diseased konjac stands infected by soft rot disease, respectively. Values are means ± standard deviation (*n* = 3). Means followed by different letters are significantly different [*p* ≤ 0.05; least significant difference (LSD) test].*

Among the three sampling sites, MD showed the highest soil sucrase activity (5.62 mg⋅g^–1^⋅day^–1^), whereas ND exhibited the highest catalase activity (1.43 μmol⋅g^–1^⋅day^–1^); however, catalase activity did not differ significantly between the MD and HD sites (Table 3). Soil urease and polyphenol oxidase activity gradually decreased with increasing degree of soft rot damage; their activity levels in the HD site were 58.4 and 63.1% lower, respectively, than those in the ND site (*p* < 0.05).

**TABLE 3:** Soil enzymatic activity in the three konjac growing sites affected by different levels of soft rot disease.

**Site**	**Sucrase activity**	**Urease activity**	**Catalase activity**	**Polyphenol oxidase activity**
	**(mg⋅g^–1^⋅day^–1^)**	**(μg⋅g^–1^⋅day^–1^)**	**(μmol⋅g^–1^⋅day^–1^)**	**(mg⋅g^–1^⋅day^–1^)**
ND	5.52 ± 0.01b	14.84 ± 0.17a	1.43 ± 0.11a	99.67 ± 0.42a
MD	5.62 ± 0.02a	7.57 ± 0.10b	1.07 ± 0.05b	50.50 ± 0.38b
HD	4.58 ± 0.01c	6.17 ± 0.13c	0.91 ± 0.09b	36.75 ± 0.57c

*ND, MD, and HD represent the non-diseased, moderately diseased, and highly diseased konjac stands infected by soft rot disease, respectively. Values are means ± standard deviation (*n* = 3). Means followed by different letter are significantly different [*p* ≤ 0.05; least significant difference (LSD) test].*

### Bacterial Taxonomic Distribution

#### Sequencing Data

**FIGURE 1:**
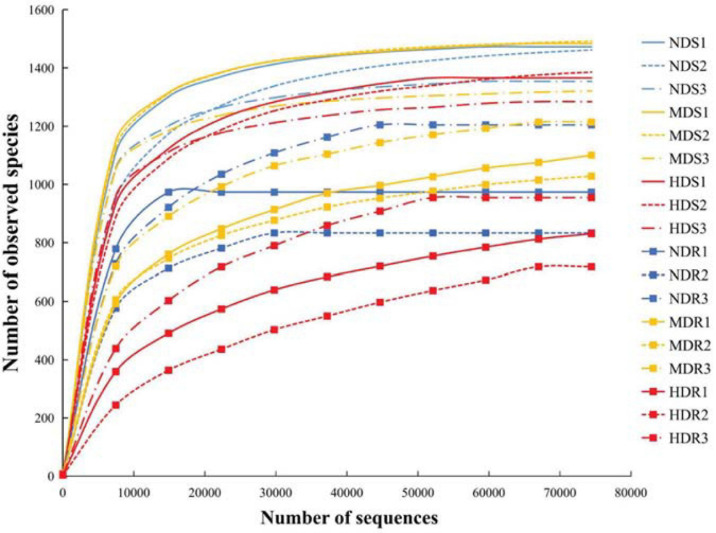
Rarefaction curves showing the relationships between the number of sequences and the number of observed species identified in the soil and root samples of konjac from the non-diseased (NDS and NDR), moderately diseased (MDS and MDR), and highly diseased (HDS and HDR) sites.

The rarefaction curves were used to indicate the volume of the sequencing data and the bacterial species richness for all the 18 samples (Song et al., 2018). The curves tended to flatten out (Figure 1), suggesting that the sequencing data can indeed represent the bacterial community in konjac soils and roots and signify the bacterial community diversity.

**FIGURE 2:**
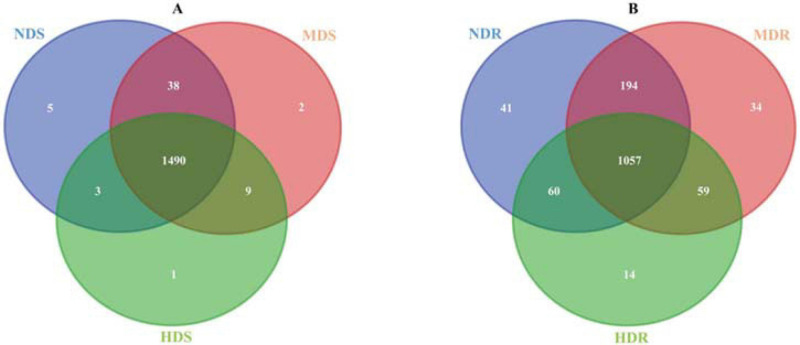
Venn diagram showing the common and unique bacterial operational taxonomic units in soil **(A)** and root **(B)** samples collected from the non-diseased (NDS and NDR), moderately diseased (MDS and MDR), and highly diseased (HDS and HDR) sites.

A total of 1,314,791 and 1,393,944 raw reads were collected from the nine soil samples and nine root samples, respectively; after paired-end assembly, 1,228,232 and 1,303,840 clean reads were obtained, respectively. In total, 987,130 and 982,774 bacterial sequences representing 12,676 and 9,235 OTUs were identified, respectively, from soil and root samples. In the soil samples from the three sampling sites (NDS, MDS, and HDS), 1,536, 1,539, and 1,503 OTUs were identified, including five, two, and one unique OTUs, respectively, with 1,490 common OTUs; the number of common OTUs was the highest (1,528) between the NDS and MDS samples. In the root samples from the three sampling sites (NDR, MDR, and HDR), fewer OTUs were identified: 1,352, 1,344, and 1,190 OTUs, respectively, with 1,057 common OTUs (Figure 2).

#### Bacterial Alpha Diversity

The soil and root samples both showed a similar tendency in the bacterial alpha diversity among the three sampling sites. One-way ANOVA showed that the bacterial species richness (ACE and Chao1) was significantly higher in the ND and MD sites than in the HD site (*p* < 0.05; Table 4). Similarly, the Shannon index suggested that the bacterial diversity decreased with increasing degree of soft rot damage, but the Simpson index presented an opposite trend. Generally, site ND had the highest bacterial species richness, whereas site HD had the lowest. Furthermore, site ND was more similar to site MD in terms of bacterial alpha diversity. Nonetheless, the Simpson index values of the soil and root samples from sites MD and HD were markedly higher than those of the samples from site ND (*p* < 0.05).

**TABLE 4:** Alpha diversity indices of soil and root bacterial communities in different konjac growing sites affected by different degrees of soft rot disease.

**Sample**		**ACE**	**Chao 1**	**Shannon index**	**Simpson index**
Soil	NDS	1451.47 ± 9.69a	1462.94 ± 2.69a	6.23 ± 0.04a	0.0063 ± 0.000042c
	MDS	1449.22 ± 12.62a	1455.63 ± 3.78a	5.98 ± 0.05b	0.0085 ± 0.000042b
	HDS	1392.90 ± 11.22b	1405.17 ± 12.99b	5.04 ± 0.01c	0.0526 ± 0.000292a
Root	NDR	1318.52 ± 1.02A	1320.88 ± 7.99A	4.80 ± 0.03A	0.0272 ± 0.000176C
	MDR	1243.72 ± 8.15B	1267.72 ± 10.50B	4.60 ± 0.01B	0.0743 ± 0.001052B
	HDR	1101.96 ± 4.57C	1098.92 ± 5.62C	2.87 ± 0.04C	0.1748 ± 0.002607A

*NDS, MDS, and HDS represent the soil samples from the non-diseased, moderately diseased, and highly diseased sites, respectively. NDR, MDR, and HDR represent the plant root samples from the non-diseased, moderately diseased, and highly diseased sites, respectively. Data are expressed as mean ± SD (*n* = 3). For each type of the samples, significant differences [*p* ≤ 0.05; least significant difference (LSD) test] among the three sampling sites are shown by different lowercase (soil) or uppercase (root) letters within each column.*

#### Bacterial Community Composition

**FIGURE 3:**
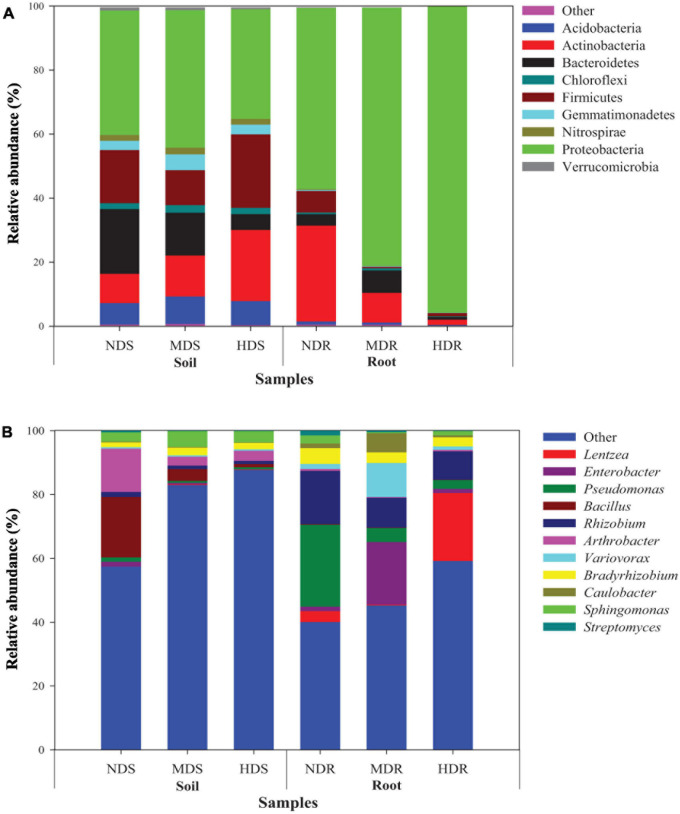
Relative abundance of dominant bacterial taxa (>1% of total sequences) in different soil and root samples. **(A)** Nine phyla and **(B)** 11 genera. NDS, MDS, and HDS represent the soil samples from the non-diseased, moderately diseased, and highly diseased sites, respectively. NDR, MDR, and HDR represent the plant root samples from the non-diseased, moderately diseased, and highly diseased sites, respectively. Data are expressed as mean ± SD (*n* = 3). For each type of samples, different lowercase (soil) or uppercase (root) letters within each group indicate significant differences among the three sampling sites [*p* ≤ 0.05; least significant difference (LSD) test].

The sequences obtained from the soil and root samples were classified into nine bacterial phyla (Figure 3A and Supplementary Table 1). In the soil samples, the most dominant bacterial phyla (relative abundance > 1%) included Proteobacteria (38.8% of all sequences), Firmicutes (16.8%), Actinobacteria (14.7%), Bacteroidetes (12.8%), Acidobacteria (7.6%), Gemmatimonadetes (3.6%), Chloroflexi (2.1%), Nitrospirae (1.9%), and Verrucomicrobia (1.2%). Among them, the relative abundance of Actinobacteria increased with increasing degree of soft rot damage, while the relative abundance of Bacteroidetes and Verrucomicrobia exhibited an opposite trend. Particularly, the abundance of Firmicutes, Acidobacteria, Chloroflexi, and Gemmatimonadetes was significantly higher (by 38.2, 12.1, 5.4, and 5.2%, respectively) in the HDS compared with NDS samples (*p* < 0.05). In contrast, Proteobacteria and Verrucomicrobia were less abundant (by 11.9% and 31.1%, respectively) in the HDS samples than in the NDS samples (*p* < 0.05). In the root samples, Proteobacteria (77.6%), Actinobacteria (13.6%), Bacteroidetes (3.8%), and Firmicutes (2.7%) were the dominant bacterial phyla. Specifically, the relative abundance of Proteobacteria increased with increasing damage degree, while the relative abundance of Acidobacteria, Actinobacteria, Gemmatimonadetes, Nitrospirae, and Verrucomicrobia showed an inverse trend. Bacteroidetes, Chloroflexi, and Firmicutes were less abundant (by 75.3, 66.0, and 84.7%, respectively) in the HDR than in NDR samples (*p* < 0.05).

At the genus level (Figure 3B and Supplementary Table 2), the relatively abundant OTUs were distributed into 11 different taxa. In the soil samples, the dominant genera (relative abundance > 1%) were *Bacillus* (7.9%), *Arthrobacter* (6.5%), *Sphingomonas* (3.8%), *Bradyrhizobium* (2.0%), and *Rhizobium* (1.2%). The relative abundance of *Enterobacter*, *Pseudomonas*, *Bacillus*, *Rhizobium*, *Arthrobacter*, *Caulobacter*, and *Streptomyces* was significantly higher in the NDS samples than in the MDS or HDS samples. However, *Bradyrhizobium* (2.4%) and *Sphingomonas* (4.9%) showed the highest relative abundance in the MDS samples. In the root samples, *Rhizobium* (11.6%), *Pseudomonas* (10.9%), *Lentzea* (8.3%), *Enterobacter* (7.4%), *Variovorax* (4.4%), *Bradyrhizobium* (3.7%), *Caulobacter* (2.7%), and *Sphingomonas* (1.4%) were predominant. The relative abundance of *Pseudomonas*, *Bacillus*, *Rhizobium*, *Bradyrhizobium*, and *Streptomyces* in the root samples decreased with increasing degree of soft rot damage and showed the lowest values in the HDR samples (2.74, 0.11, 8.90, 2.87, and 0.11%, respectively). Compared with those in the NDR samples, *Lentzea* was 6.3-fold more abundant in the HDR samples, whereas *Enterobacter*, *Variovorax*, and *Caulobacter* were 13.6-, 6.8-, and 4.1-fold more abundant in the MDR samples, respectively. Moreover, the relative abundance of *Arthrobacter* and *Sphingomonas* in the HDR samples was 24.6% and 48.6% lower than in the NDR samples (*p* < 0.05).

**FIGURE 4:**
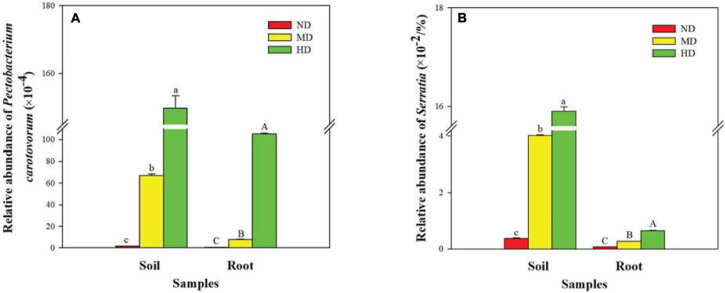
Relative abundance of soft rot pathogens in different soil and root samples. **(A)** *Pectobacterium carotovorum* and **(B)** *Serratia*. ND, MD, and HD represent the non-diseased, moderately diseased, and highly diseased sites, respectively. Data are expressed as mean ± SD (*n* = 3). For each type of samples, different lowercase (soil) or uppercase (root) letters within each group indicate significant differences among the three sampling sites [*p* ≤ 0.05; least significant difference (LSD) test].

As expected, bacterial sequences of soft rot pathogens were identified in both soil and root samples (Figures 4A,B). The relative abundance of *Pectobacterium carotovorum* and *Serratia* increased with increasing damage degree and reached the highest values in the HD site (HDS: 1.5 and 0.16%, respectively; HDR: 1.0 and 0.01%, respectively).

### Factors Associated With Bacterial Community Structure

**FIGURE 5:**
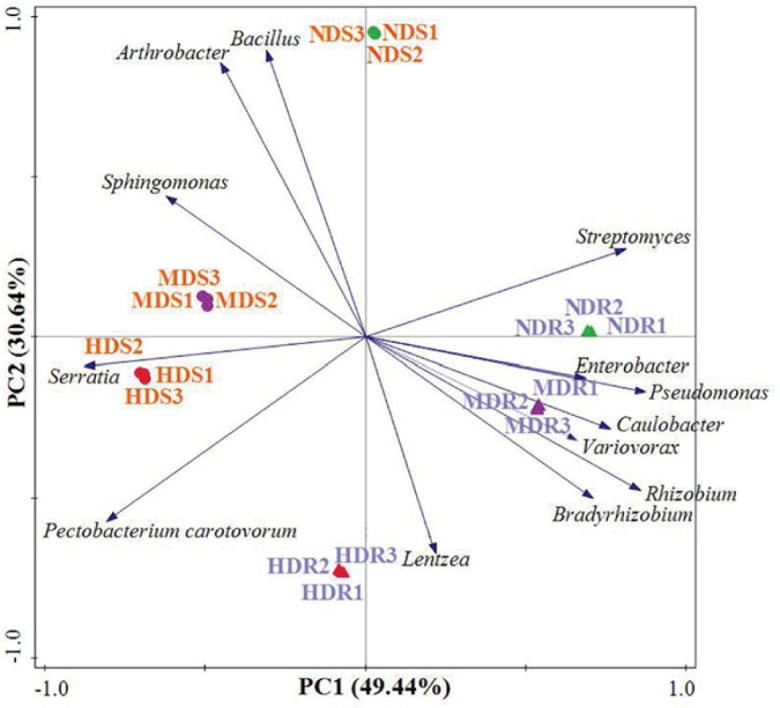
Principal component analysis of the dominant and pathogenic bacterial operational taxonomic units (genera) recovered from the root and soil samples in different konjac growing sites. NDS, MDS, and HDS represent the soil samples from the non-diseased, moderately diseased, and highly diseased sites, respectively. NDR, MDR, and HDR represent the plant root samples from the non-diseased, moderately diseased, and highly diseased sites, respectively.

The PCA plot based on the relative abundance of the 11 dominant and two pathogenic bacterial genera shows a separation between soil and root samples, as well as between NDS and MDS/HDS samples, or between the NDR/MDR and HDR samples on the first component axis, which explained 49.4% of the total variation (Figure 5). The second component explained 30.6% of the total variation. The component loading plot shows groups of OTU associated with the different konjac samples. In the root samples, a large group of OTUs, including those belonging to genera *Enterobacter*, *Pseudomonas*, *Caulobacter*, *Variovorax*, *Rhizobium*, and *Bradyrhizobium*, were associated with the MDR samples. Moreover, OTUs belonging to *Streptomyces*, *Enterobacter*, and *Pseudomonas* were associated with the NDR samples, while those from *Lentzea* and *P. carotovorum* were associated with the HDR samples. In the soil samples, a large group of OTUs, including those belonging to genera *Bacillus*, *Arthrobacter*, and *Sphingomonas*, were associated with the NDS samples. Additionally, OTUs from *Sphingomonas* and *Serratia* were associated with the MDS samples, while those from *Serratia* and *P. carotovorum* were associated with the HDS samples (Figure 5).

**FIGURE 6:**
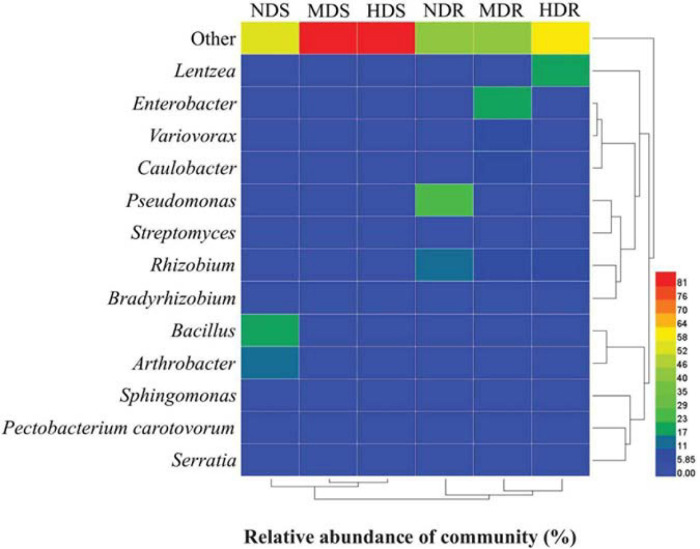
Heatmap of bacterial operational taxonomic units in soil and root samples of konjac from the non-diseased (NDS and NDR), moderately diseased (MDS and MDR), and highly diseased (HDS and HDR) sites.

**FIGURE 7:**
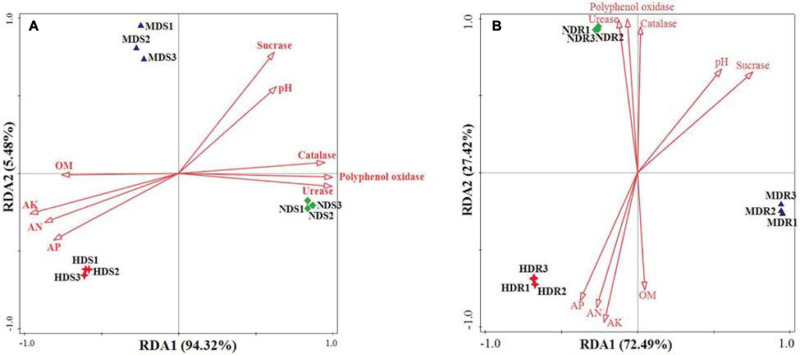
Redundancy analysis revealing the effects of different degrees of soft rot disease on soil variables and bacterial community structure (genus level) in the soil **(A)** and root **(B)** samples of konjac from the non-diseased (NDS and NDR), moderately diseased (MDS and MDR), and highly diseased (HDS and HDR) sites. OM, organic matter; AN, alkaline-hydrolyzable nitrogen; AK, available potassium; and AP, available phosphorus.

The OTU data generated a high resolution for distinguishing the three sampling sites in terms of bacterial communities. All the bacterial communities recovered from each site clustered together with high identity (Figure 6). The clustering analysis showed a clear separation between the three sampling sites, indicating that different sites had distinctive characteristics and significantly influenced the bacterial communities. Generally, the MD and HD sites were more similar to each other than to the ND site in terms of the bacterial community structure in both soil and root samples. Furthermore, the RDA results showed that, in the soil samples, RDA1 separated NDS from MDS and HDS, whereas RDA2 separated MDS from HDS (Figure 7A); in the root samples, RDA1 separated MDR from NDR and HDR, whereas RDA2 separated NDR from HDR (Figure 7B). Soil AK, sucrase activity, and urease activity showed the largest effects on the bacterial community structure in both soil and root samples.

## Discussion

In the present study, soil pH and nutrient levels changed at a small site scale due to the development of konjac soft rot under a *R. pseudoacacia* plantation. Specifically, soil pH significantly decreased with increasing degree of konjac soft rot, which is in agreement with the finding of Wu et al. (2017) that soil pH was higher in healthy soils than in diseased soils with continuously cropped konjac plants infected by *Pectobacterium* spp. The lower pH after soft rot development could be explained by the accumulation of organic acid through anaerobic decomposition and the associated environmental variation. For instance, the variation in soil conditions could be caused by weathering and biotic factors such as mycorrhizal development (Akema and Futai, 2005). Moreover, soil OM, AN, AP, and AK contents were significantly higher in the HD site than in the ND konjac site, which is probably attributed to increased OM decomposition, decreased carbon uptake, and reduced utilization of nutrients (N, P, and K) under the influence of soft rot disease.

Soil enzymatic activity is regarded as a crucial indicator of soil quality and agroecological stability (Acosta-Martinez et al., 2018). Sucrase catalyzes the hydrolysis of sucrose to produce fructose and glucose (Frankeberger and Johanson, 1983), while catalase catalyzes the decomposition of H_2_O_2_ into O_2_ and H_2_O (Kou et al., 2018). Here, soil sucrase and catalase activity exhibited a remarkable decrease in the HD site compared with the ND site of konjac. This result concurs with the findings from a previous study on notoginseng (*Panax notoginseng*) under no-till cultivation (You et al., 2014), indicating that an increase in the enzymatic activity of sucrase and catalase may be a response to soft rot disease and may enhance the protective effect on plant cells. Urease catalyzes the conversion of urea to ammonia and carbon dioxide (Phang et al., 2018), while polyphenol oxidase is mainly involved in the metabolism of aromatic organic carbon (Li et al., 2015). Soil urease and polyphenol oxidase activity exhibited a remarkable and continuous decrease with increasing incidence of konjac soft rot, which may be not advantageous to nitrogen utilization and carbon metabolism of plants affected by soft rot.

Proteobacteria and Actinobacteria, the most abundant phyla in various agroecosystems (Farmer et al., 2016; Huang, 2018), were the top two predominant bacterial phyla in both soil and root samples of konjac. And the relative abundance of Proteobacteria in the MD site (soil and root) and HD site (root) was significantly higher than that in the ND site. Similar results were obtained in watermelon (*Citrullus lanatus*) where the relative abundance of Proteobacteria in the soil of wilted watermelon was higher than that in healthy soil under both rotation and continuous cropping systems (Liang, 2018). However, these results contradict those of Lin et al. (2019), who reported that the abundance of Proteobacteria was negatively correlated with the incidence of banana (*Musa nana*) wilt under a banana–sugarcane rotation system (Lin et al., 2019). These conflicting results suggest that the relationship between the phylum Proteobacteria and the soft rot of konjac is dependent on the indirect effects of other factors such as various microbial taxa, plant developmental stage, and environmental conditions.

At the genus level, the relative abundance of *Pseudomonas* significantly decreased with increased incidence of konjac soft rot. Liu (2014) also reported that the abundance of *Pseudomonas*, which is closely associated with healthy plant growth, was significantly higher in the rhizosphere and non-rhizosphere soil of healthy plants than in diseased plants of ginger (*Zingiber officinale*; Liu, 2014). The relative abundance of *Bacillus* was significantly higher in the soil and root endosphere of the ND konjac plants than in the MD and HD plants, suggesting a vital role of *Bacillus* in healthy konjac growth. A previous study has demonstrated that *Bacillus* spp. are antagonistic to various potential phytopathogens including bacterial pathogens, fungal pathogens, systemic viruses, and root-knot nematodes (Kloepper et al., 2004). Similarly, the relative abundance of *Rhizobium* and *Streptomyces* was higher in the soil and root endosphere of healthy konjac plants. Owing to their high colonization ability, *Rhizobium* spp. could also be used in non-legumes for growth promotion (Qureshi et al., 2013). Moreover, *Streptomyces* are prolific producers of natural products with biological activities, such as antibiotics, which play a positive role in the plant rhizosphere microecosystem and control soil-borne pathogens (Jain and Jain, 2007; Xue et al., 2013).

In support of our previous culture-based study (He et al., 2018), here, we found that culturable dominant bacteria, including *Bacillus thuringiensis*, *Rhizobium radiobacter*, and *Streptomyces cellulosae*, were highly associated with the ND site, although it is difficult to determine the exact abundance of each species by the plate counting method. Additionally, the relative abundance of genera *Bradyrhizobium* and *Sphingomonas* was higher in the root endosphere of MD and HD konjac plants. *Bradyrhizobium* spp. and *Sphingomonas* spp. are reported to have high nitrogen-fixing ability and may enhance the drought and salinity tolerance of plants (Sun et al., 2010; Meng, 2016; Oliveira et al., 2017). These potentially beneficial bacteria, which were abundant in the root zone of ND konjac plants, could have enhanced their resistance to soft rot disease by producing antibacterial substances and inducing systemic resistance. Indeed, *Streptomyces*, *Rhizobium*, and *Bacillus* strains were previously used as biocontrol agents to protect konjac from soft rot (He et al., 2015a, b).

Estimations of the bacterial community composition in the soil and root endosphere of a crop could provide new opportunities for exploring the potential antagonistic microbes for suppression of soil-borne plant pathogens (Song et al., 2018). *P. carotovorum* is a bacterial pathogen causing soft rot disease in diverse plant species such as konjac (Wu et al., 2011a), pinellia (*Pinellia ternata*; Hu et al., 2008), cucumber (*Cucumis sativus*; Nazerian et al., 2011), and potato (*Solanum tuberosum*; Naas et al., 2018). In our study, the relative abundance of *P. carotovorum* was 1.498 and 1.051% in the soil and root endosphere of the HD konjac plants, respectively. These values were slightly higher than the relative abundance of *Serratia*, which is another group of potential pathogens causing soft rot disease (Gillis et al., 2013; Abu-Obeid et al., 2017) in the HD konjac plants (0.159 and 0.006%, respectively). *Serratia* spp. have been reported to cause soft rot of onion (*Allium cepa*; Lv et al., 2013) and pepper (*Capsicum annuum*; Gillis et al., 2013). As expected, the relative abundance of *P. carotovorum* and *Serratia* was lower in the ND site than in the MD and HD sites of konjac. Changes in the abundance of these pathogens inhabiting the soil and root endosphere of konjac plants may be associated with the incidence of soft rot.

The occurrence of disease pathogens and pests can cause shifts in root-associated bacterial communities (Kim et al., 2016; Silva et al., 2016). In the present study, the bacterial taxonomic distribution indicated that the relative abundance of dominant taxa was especially affected by different degrees of soft rot damage. Moreover, specific taxa could be related to the characteristics of distinct konjac growing sites. For instance, *Bacillus*, *Streptomyces*, *Enterobacter*, and *Pseudomonas* were associated with the ND site; *Sphingomonas*, *Caulobacter*, and *Variovorax* were associated with the MD site; and *Serratia*, *P. carotovorum*, and *Lentzea* were associated with the HD site. Therefore, these dominant genera may be used as indicators to assess the degree of damage in konjac plants due to soft rot disease. Furthermore, the bacterial alpha diversity indices were significantly lower in the MD and HD konjac sites. Zhang et al. (2013) also found that bacterial Shannon–Wiener and McIntosh indices were much lower in the soil of garlic (*Allium sativum*) infected by root rot disease than in those of healthy plants. The results clearly demonstrated that the infection of konjac plants due to *P. carotovorum* and *Serratia* altered the root-associated bacterial community structure. The effect of potentially beneficial bacteria on the resistance of konjac plants against soft rot merits further study.

## Conclusion

In the present study, three sampling sites were investigated to explore the effects of soft rot damage on bacterial communities associated with the roots of konjac plants growing under a pure *R. pseudoacacia* plantation. Different degrees of soft rot disease altered soil physicochemical properties, with a large decrease in soil pH and an increase in soil OM, alkaline-hydrolyzable nitrogen, AP, and AK contents. Meanwhile, soil enzymatic activity was decreased, while the abundance and alpha diversity of root-associated bacteria were reduced due to soft rot damage of konjac plants. In conclusion, soft rot disease caused remarkable shifts in the community structure of root-associated bacteria of konjac mainly by altering the levels of soil AK, sucrase activity, and urease activity. Quantification of the dominant bacterial taxa provides an approach to predict the degree of damage due to konjac soft rot.

## Data Availability Statement

The datasets presented in this study can be found in online repositories. The names of the repository/repositories and accession number(s) can be found below: https://www.ncbi.nlm.nih.gov/, PRJNA623222. All raw sequences were submitted to the Sequence Read Archive of the National Center for Biotechnology Information database under the accession number from SRR11490401 to SRR11490418.

## Author Contributions

FH conceived, designed, performed the experiments, analyzed the data, and wrote the manuscript.

## Conflict of Interest

The author declares that the research was conducted in the absence of any commercial or financial relationships that could be construed as a potential conflict of interest.

## Footnotes

1 http://www.r-project.org/

## Supplementary Material

Supplementary Figure 1 Growth conditions of konjac plants in the non-diseased (ND; **A**), moderately diseased (MD; **B**), and highly diseased (HD; **C**) sampling sites under a pure *Robinia pseudoacacia* plantation.

Supplementary Table 1 Significant differences in the relative abundance of major bacterial phyla among three soil and three root samples of konjac collected from the non-diseased (NDS and NDR), moderately diseased (MDS and MDR), and highly diseased (HDS and HDR) sites. Different lowercase and uppercase letters indicate significant differences among the soil and root samples from different sampling sites, respectively (*P* ≤ 0.05; LSD test).

Supplementary Table 2 Significant differences in the relative abundance of dominant bacterial genera among three soil and three root samples of konjac collected from the non-diseased (NDS and NDR), moderately diseased (MDS and MDR), and highly diseased (HDS and HDR) sites. Different lowercase and uppercase letters indicate significant differences among the soil and root samples from different sampling sites, respectively (*P* ≤ 0.05; LSD test).
