# Supplementary material for: Response of Root-Associated Bacterial Communities to Different Degrees of Soft Rot Damage in Amorphophallus konjac Under a Robinia pseudoacacia Plantation
Source: Front Microbiol. 2021 Jul 8;12:652758. doi: 10.3389/fmicb.2021.652758 (PMC8297708; doi:10.3389/fmicb.2021.652758)
Supplement: Supplementary Figure 1 — Growth conditions of konjac plants in the non-diseased (ND; A), moderately diseased (MD; B), and highly diseased (HD; C) sampling sites under a pure Robinia pseudoacacia plantation. [file Table_1.doc]

**Table S1** | Significant differences in the relative abundance of major bacterial phyla among three soil and three root samples of konjac collected from the non-diseased (NDS and NDR), moderately diseased (MDS and MDR), and highly diseased (HDS and HDR) sites.

| Taxonomy | Soil | | | Root | | |
| --- | --- | --- | --- | --- | --- | --- |
|  | NDS | MDS | HDS | NDR | MDR | HDR |
| Acidobacteria | c | a | b | A | B | C |
| Actinobacteria | c | b | a | A | B | C |
| Bacteroidetes | a | b | c | B | A | C |
| Chloroflexi | c | a | b | B | A | C |
| Firmicutes | b | c | a | A | C | B |
| Gemmatimonadetes | c | a | b | A | B | C |
| Nitrospirae | b | a | b | A | B | C |
| Proteobacteria | b | a | c | C | B | A |
| Verrucomicrobia | a | b | c | A | B | C |
| Other | b | a | c | A | A | B |

Different lowercase and uppercase letters indicate significant differences among the soil and root samples from different sampling sites, respectively (*P* ≤ 0.05; LSD test).

**Table S2** | Significant differences in the relative abundance of dominant bacterial genera among three soil and three root samples of konjac collected from the non-diseased (NDS and NDR), moderately diseased (MDS and MDR), and highly diseased (HDS and HDR) sites.

| Taxonomy | Soil | | | Root | | |
| --- | --- | --- | --- | --- | --- | --- |
|  | NDS | MDS | HDS | NDR | MDR | HDR |
| *Lentzea* | c | a | b | B | C | A |
| *Enterobacter* | a | b | c | B | A | B |
| *Pseudomonas* | a | b | b | A | B | C |
| *Bacillus* | a | b | c | A | B | B |
| *Rhizobium* | a | b | b | A | B | B |
| *Arthrobacter* | a | b | b | A | C | B |
| *Variovorax* | a | a | a | B | A | C |
| *Bradyrhizobium* | c | a | b | A | B | C |
| *Caulobacter* | a | b | c | B | A | C |
| *Sphingomonas* | c | a | b | A | C | B |
| *Streptomyces* | a | b | b | A | B | C |
| Other | c | b | a | C | B | A |

Different lowercase and uppercase letters indicate significant differences among the soil and root samples from different sampling sites, respectively (*P* ≤ 0.05; LSD test).


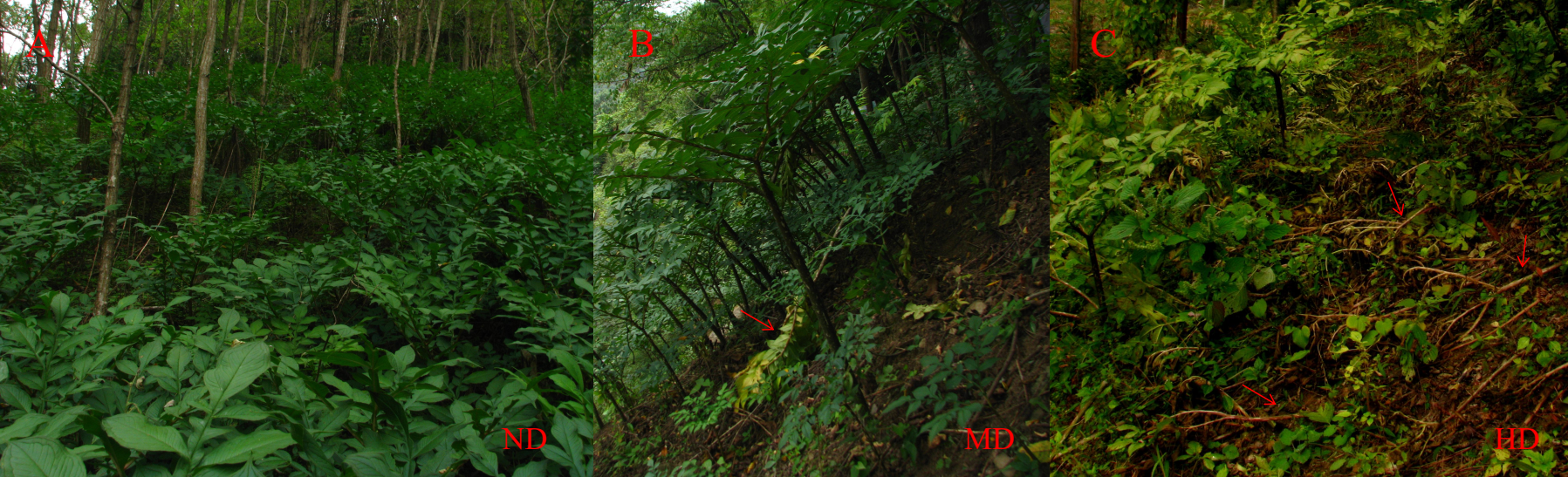


**FIGURE S1** | Growth conditions of konjac plants in the non-diseased (ND; A), moderately diseased (MD; B), and highly diseased (HD; C) sampling sites under a pure *Robinia pseudoacacia* plantation.
